# Contrasting patterns of genetic variation in core and peripheral populations of highly outcrossing and wind pollinated forest tree species

**DOI:** 10.1093/aobpla/plw054

**Published:** 2016-08-06

**Authors:** Błażej Wójkiewicz, Monika Litkowiec, Witold Wachowiak

**Affiliations:** 1 Institute of Dendrology, Polish Academy of Sciences, Parkowa 5, Kórnik 62-035, Poland; 2 Faculty of Biology, Adam Mickiewicz University, Institute of Environmental Biology, Umultowska 89, Poznań 61-614, Poland

**Keywords:** Demographic history, genetic structure, glacial refugia, phylogeography, *Pinus sylvestris*, population history, recolonization

## Abstract

Gene flow tends to have a homogenising effect on a species’ background genetic variation over large geographical areas. However, it is usually unknown to what extent the genetic structure of populations is influenced by gene exchange between core and peripheral populations that may represent stands of different evolutionary and demographic history. In this study, we looked at the patterns of population differentiation in Scots pine—a highly outcrossing and wind pollinated conifer species that forms large ecosystems of great ecological and economic importance in Europe and Asia. A set of 13 polymorphic nuclear microsatellite loci was analysed to infer the genetic relationships among 24 populations (676 individuals) from Europe and Asia Minor. The study included specimens from the primary continuous range and from isolated, marginal stands that are considered to be autochthonous populations representative of the species’ putative refugial areas. Despite their presumably different histories, a similar level of genetic variation and no evidence of a population bottleneck was found across the populations. Differentiation among populations was relatively low (average *F*_ST _= 0.035); however, the population structure was not homogenous, which was clearly evident from the allelic frequency spectra and Bayesian assignment analysis. Significant differentiation over short geographical distances was observed between isolated populations within the Iberian and Anatolian Peninsulas (Asia Minor), which contrasted with the absence of genetic differentiation observed between distant populations e.g., between central and northern Europe. The analysed populations were assigned to several groups that corresponded to the geographical regions of their occurrence. These results will be useful in genetics studies in Scots pine that aim to link nucleotide and phenotypic variation across the species distribution range and for development of sustainable breeding and management programs.

## Abstract (precis)

This study of nuclear microsatellite loci demonstrates genetic relationships between twenty four Scots pine populations from Europe and Asia Minor. The analysed populations were assigned to several groups that corresponded to the geographical regions of their occurrence. Significant differentiation over short geographical distances was observed between isolated populations within the Iberian and Anatolian Peninsulas (Asia Minor), which contrasted with the absence of genetic differentiation between distant populations, for instance in central and northern Europe. Despite the signs of isolation found in peripheral stands, a similar level of genetic variation and no evidence of a recent bottleneck was found across the populations.

## Introduction

Demographic and evolutionary processes interplay to shape genetic variation that is crucial to maintaining species’ adaptive responses to changing environments. Genetic variation among plant populations in the Northern Hemisphere has been shaped by range shifts and recolonization following the last glacial maximum (25–18 000 years ago) (Petit *et al.* 2003; Nosil and Feder 2013). The assessment of the genetic relationships among natural populations across the species distribution is important for tree management and for breeding and gene conservation programs, particularly in the face of ongoing environmental changes (Savolainen *et al.* 2011).

Forest trees are known to form large, wind pollinated populations and maintain a high level of genetic and phenotypic variation in comparison to other plant species (Petit and Hampe 2006). High diversity ensures that these long-lived organisms can survive and evolve under changing environmental conditions. Studies of historical processes such as population size fluctuations and geographical range shifts are needed to better understand the effect of demographic factors that influence background genetic variation and to effectively contrast neutral variation with that resulting from natural selection (Luikart *et al.* 2003; Li *et al.* 2012). However, currently available genetic data still lack sufficient information to fully reflect the usually complex demographic history of many forest tree species. For highly outcrossing and wind pollinated species, gene flow is supposed to have homogenising effects on the background, neutral genetic variation between populations over large geographical distances. However, its effect on the distribution of genetic variation between populations from core range vs. marginal populations of the species is not that clear, especially for long-lived temperate forest tree species.

In the present study, we focused on Scots pine (*Pinus sylvestris*), which is one of the most ecologically and economically important forest-forming tree species in Eurasia. Some former phylogeographic studies of this pine based on isozyme polymorphisms, organelle DNA and palynological records have shown that the most abundant populations of Scots pine survived the cold periods of the Pleistocene within southern Eurasia in the Iberian, Apennine and Balkan Peninsulas and in the Anatolian and Caucasus Mountains (Willis *et al.* 1998; Willis and van Andel 2004; Cheddadi *et al.* 2006; Naydenov *et al.* 2007; Pyhäjärvi *et al.* 2008; Soto *et al.* 2010; Buchovska *et al.* 2013). The Alps, the Carpathians and Moscow are considered refugial areas for other cold tolerant conifer species including Norway spruce (*Picea abies*) (Tollefsrud *et al.* 2008, 2009). Although there are strong indications regarding the location of some putative refugial stands, it is less clear how migration and gene flow have influenced the patterns of genetic variation at neutral gene markers across the present range of the species. Consequently, the relationships among the gene pools of populations from core and peripheral distribution are not well elucidated.

The aim of this study was to assess the genetic variation and structure of Scots pine populations from central and north Europe in relation to its marginal populations from the European and south-west Asiatic refugial areas based on the analysis of nuclear simple sequence repeat (*n*SSR) markers. Nuclear markers in pines are distributed by seeds and pollen and could potentially be dispersed at large geographical distances. Therefore, the identification of populations with divergent genetic backgrounds could suggest the existence of distinct populations that do not share a recent history. To our knowledge, this species has not yet been investigated on such a large geographical scale using this type of neutral marker. In this study, we used a set of 13 *n*SSR loci to examine genetic diversity and test for the existence of populations of different origin. We aimed to check if there is any difference between core and peripheral populations considering the presumably homogenising effect of long distance gene flow. Information regarding the genetic relationships among populations is particularly important to advance studies of the genetic architecture of observed variation in phenotypic traits that have been shaped by selection and local adaptation within the Scots pine distribution.

## Methods

### Plant material, DNA extraction and microsatellite genotyping

**Figure 1.**
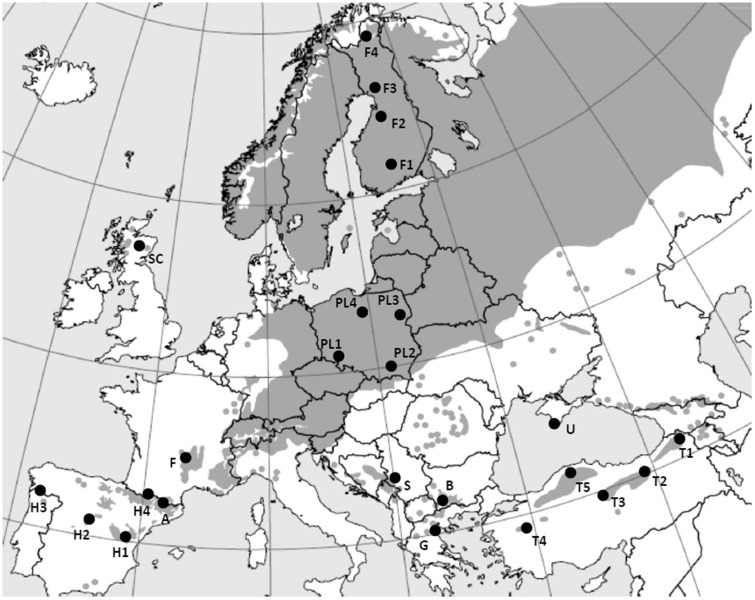
Geographic location of Scots pine populations included in this study. Acronyms and geographic coordinates of the populations are listed in **Supporting Information—Table S1**.

The study comprised 24 Scots pine populations (Fig. 1), 8 of which were from the core continuous species distribution from central and northern Europe. The rest of the populations analysed were collected from isolated, peripheral stands on the Iberian and Anatolian Peninsulas, the Massif Central of France, Scotland and the Balkans [**see Supporting Information—Table S1**]. The number of samples per population varied from 22 to 49 individuals with a total of 676 individuals (Table 1). Genomic DNA was extracted from needles using a CTAB protocol (Dumolin *et al.* 1995). The initial set of 22 nuclear microsatellite markers originally identified in pine species (Soranzo *et al.* 1998; Elsik *et al.* 2000; Liewlaksaneeyanawin *et al.* 2004; Sebastiani *et al.* 2012) were screened for their ability to provide repeatable, high quality results, sufficient polymorphism and unambiguous allele binding. The final set of loci used in this study included 13 *n*SSRs that provided high-quality amplification products. DNA amplification was carried out in three multiplex reactions including loci psyl2, psyl18, psyl25, psyl36, psyl42, psyl44 and psyl57 (multiplex 1); Spag7.14, PtTX2146, PtTX3107 and Spac11.4 (multiplex 2) and PtTX3025 and PtTX4011 (multiplex 3). The PCR for each sample was conducted in a total volume of 10 μL containing 5 μL of Qiagen Multiplex Master Mix (2×), 0.2 μL of primer mix (20 μM), 1 µL of Q-Solution (5×), 0.8 μL RNase-free water and 3 μL of DNA 84 template (approximately 10–20 ng). The following PCR amplification conditions were used: multiplex 1, initial denaturation at 95 °C for 15 min, 38 cycles of denaturation at 94 °C for 30 s, annealing at 57 °C for 90 s, extension at 72 °C for 90 s and final extension at 72 °C for 10 min; multiplex 2, initial denaturation at 95 °C for 15 min, 30 cycles of denaturation at 94 °C for 30 s, annealing at 55 °C for 90 s, extension at 72 °C for 90 s and final extension at 72 °C for 10 min; multiplex 3, initial denaturation at 95 °C for 15 min, 10 cycles of denaturation at 94 °C for 30 s, annealing at 60 °C for 40 s with temperature decreasing by 1 degree per cycle, extension at 72 °C for 90 s, 36 cycles of denaturation at 94 °C for 30 s, annealing at 50 °C for 40 s, extension at 72 °C for 90 s and final extension at 72 °C for 10 min. The fluorescently labelled PCR products, along with a size standard (GeneScan 500 LIZ), were separated on a capillary sequencer ABI 3130 (Thermo Fisher Scientific, Waltham, USA). The allele’s size was determined using GeneMapper software (ver. 4.0; Life Technologies).

**Table 1.** Genetic variation of Scots pine populations based on thirteen polymorphic nSSR loci. Nr and pop, population number and acronyms with reference to Fig. 1 and **Supporting Information — Table S1**, *N*, number of analyzed individuals; Np, the mean number of alleles per population; Ne, mean number of effective alleles per population; *A*_R22_, allelic richness for a minimum sample size of 22 individuals; Pa, mean number of private alleles per population; Gd, gene diversity; Ho, observed heterozygosity; He, unbiased expected heterozygosity; *F*_is_, inbreeding coefficient (*P < 0.001); *F*_isNull_, inbreeding coefficient with null allele correction.

Nr.	Pop	*N*	Np	Ne	A_R22_	Pa	Gd	Ho	He	*F*_is_	*F*_isNull_
1.	T1	25	5.46	3.39	5.19	0.00	0.53	0.477	0.515	0.09*	0.04 (0–0.12)
2.	T2	25	5.77	3.31	5.37	0.00	0.51	0.423	0.500	0.17*	0.07 (0–0.15)
3.	T3	25	5.54	2.91	5.33	0.08	0.52	0.456	0.507	0.12*	0.07 (0–0.17)
4.	T4	25	5.77	3.16	5.47	0.08	0.52	0.418	0.502	0.19*	0.08 (0–0.15)
5.	T5	25	6.23	3.41	5.83	0.23	0.53	0.483	0.521	0.09*	0.08 (0–0.15)
6.	U	25	4.69	3.10	5.59	0.15	0.50	0.377	0.516	0.28*	0.07 (0–0.13)
7.	G	31	6.62	3.45	5.85	0.00	0.54	0.494	0.536	0.09*	0.06 (0–0.06)
8.	B	25	6.31	3.62	5.87	0.00	0.54	0.490	0.530	0.09*	0.02 (0–0.08)
9.	S	26	6.15	3.49	5.68	0.00	0.53	0.481	0.515	0.08*	0.03 (0–0.13)
10.	H1	29	7.15	3.50	5.84	0.15	0.55	0.434	0.505	0.15*	0.06 (0–0.17)
11.	H2	31	7.00	3.25	5.27	0.15	0.49	0.370	0.439	0.18*	0.07 (0–0.15)
12.	H3	29	6.31	3.36	5.52	0.08	0.51	0.418	0.489	0.16*	0.07 (0–0.10)
13.	A	32	5.92	3.23	5.26	0.08	0.50	0.440	0.487	0.12*	0.05 (0–0.08)
14.	H4	32	5.92	3.25	5.31	0.00	0.49	0.456	0.481	0.07*	0.03 (0–0.06)
15.	FR	25	6.31	3.51	5.93	0.08	0.50	0.444	0.487	0.11*	0.02 (0–0.12)
16.	SC	39	6.31	3.01	5.34	0.23	0.50	0.437	0.491	0.12*	0.04 (0–0.10)
17.	PL1	33	6.54	3.84	6.21	0.00	0.52	0.467	0.496	0.07*	0.04 (0–0.10)
18.	PL2	45	5.85	3.70	5.66	0.00	0.51	0.456	0.536	0.12*	0.03 (0–0.08)
19.	PL3	22	5.46	3.22	5.28	0.08	0.45	0.425	0.483	0.14*	0.03 (0–0.08)
20.	PL4	28	6.23	3.59	5.65	0.00	0.48	0.409	0.475	0.13*	0.04 (0–0.07)
21.	F1	25	6.38	3.47	5.79	0.00	0.50	0.471	0.489	0.08*	0.02 (0–0.06)
22.	F2	25	6.38	3.69	5.85	0.08	0.50	0.491	0.490	0.02*	0.02 (0–0.05)
23.	F3	25	5.77	3.12	5.43	0.08	0.49	0.452	0.500	0.11*	0.03 (0–0.08)
24.	F4	24	5.77	3.16	5.42	0.00	0.48	0.458	0.470	0.06*	0.02 (0–0.07)
**Mean**		28.2	6.08	3.36	5.58	0.06	0.51	0.447	0.498	0.12*	0.04 (0–0.07)

### Allelic diversity and within-population genetic variation

Genotypic disequilibrium between pairs of loci was tested at the single population level and across all populations with a Fisher’s exact test using ARLEQUIN 3.11 (Excoffier and Lischer 2010). The allelic diversity of the studied loci and within-population genetic variation were estimated based on the following parameters: the number of alleles per locus (Al), the mean number of alleles per population (Np), the mean number of effective alleles per population (Ne), the mean number of private alleles per population (Pa), the observed heterozygosity (Ho) and the unbiased expected heterozygosity (He), all of which were computed using GenAlEx 6 (Peakall and Smouse 2006). In accordance with earlier studies that showed that microsatellites are known to be susceptible to genotyping errors (Guichoux *et al.* 2011), the null allele frequency for each loci was calculated using the EM algorithm with FreeNA software (Chapuis and Estoup 2007). We used FSTAT v 2.9.3 (Goudet 2001) to estimate gene diversity (Gd), rarefied allelic richness (A_R22_) for a minimum sample size of 22 individuals and inbreeding coefficient values (*F*_is_). The deviation of genotypic frequencies from Hardy–Weinberg equilibrium (HWE) were also identified utilizing the inbreeding coefficients (*F*_is_; Weir and Cockerham 1984) with a correction for null alleles (*F*_isNull_) for each population using the Bayesian method implemented in INEST 2.0 software (Chybicki 2015). The evaluation was performed using the IIM model with 100 000 MCMC iterations, storing every 100th value and with a burn-in period of 10 000. A Bayesian procedure based on the Deviance Information Criterion (DIC) was used to determine the statistical significance of the inbreeding component by comparing the full model with the random mating model (under the assumption *F*_is _= 0).

### Genetic differentiation between populations

To estimate the proportion of the total genetic variation due to differentiation among populations, an Analysis of Molecular Variance (AMOVA) based on two distance methods (*F*_ST_ and *R*_ST_) was conducted using ARLEQUIN 3.11. Moreover, due to the presence of null alleles, the global, pairwise and within-geographic regions *F*_ST_ were calculated using FreeNA software. FreeNA applies the ENA (Excluding Null Alleles, *F*_ST_ENA) correction method to effectively correct for the positive bias induced by the presence of null alleles in the *F*_ST_ estimation. Bootstrap 95 % confidence intervals (CI) were calculated for the global *F*_ST_ENA values using 2000 replicates across the loci. The statistical significance of the *F*_ST_ values was verified with ARLEQUIN 3.11.

To evaluate the ability of the stepwise mutation model (SMM) to differentiate among populations and geographical regions, which in turn indicates whether phylogeographical structures exist, the computed *F*_ST_ and *R*_ST_ were compared. To test whether the difference between values of *R*_ST_ and permuted p*R*_ST_ (which corresponds to *F*_ST_) was significant, the permutation test proposed by Hardy *et al.* (2003) was implemented in the program SpaGeDi 1.3d (Hardy and Vekemans 2002).

The genetic population structure (in the case of microsatellite markers) can arise due to isolation by distance (IBD), range expansions, diffusion of genes through space in migratory events and/or allelic surfing (Diniz-Filho *et al.* 2013). Because of that a Mantel test (1967) was applied to evaluate spatial processes driving populations structure by comparing the matrixes of pairwise geographic (logarithmic scale) and pairwise genetic (measured as *F*_ST/_(1−*F*_ST_)) distances. The statistical significance of the correlation was calculated for all populations and sets of populations located along latitudinal and longitudinal transects using 9999 permutations with GenAlEx 6.

### Population clustering and phylogenetic relationships

Principal Coordinates Analysis (PCoA) was applied to visualize the patterns of the genetic structure of the populations using a pairwise F_ST_ENA matrix and GenAlEx 6 software. Phylogenetic relationships between the populations were investigated using POPTREEW (Takezaki *et al.* 2014). The phylogenetic tree was constructed from allele frequency data using the neighbour-joining (NJ) method. This method allows faithful depiction of genetic structure for some populations that have an isolation-by-distance population structure (Kalinowski 2009). Nei's standard genetic distance (*D*_ST_) (Nei 1972) was chosen as a distance measure for the construction of the phylogeny. The statistical robustness of the branches was evaluated with 1000 bootstrap replicates.

The assignment of individuals and populations to genetically distinct groups was conducted using the Bayesian clustering method with the software STRUCTURE 2.3.4 (Pritchard *et al.* 2000; Falush *et al.* 2003; Hubisz *et al.* 2009). This program uses a Markov chain Monte Carlo (MCMC) algorithm to assign individuals to a given number of genetic clusters (*K*) without considering sampling origins and assuming that each cluster is in optimal Hardy–Weinberg (H–W) and linkage equilibrium (LE). The correlated allele frequencies and admixture model used allowed for mixed recent ancestry of individuals and assigned the proportion of the genome of each individual to the inferred clusters. Moreover, because all the microsatellite loci used in this study were affected by null alleles (see Results section), the recessive alleles option was chosen. Twenty independent runs were performed for each *K*, from *K* = 1 to 24, with burn-in lengths of 500 000 and 100 000 iterations. The probability distributions of the data (Ln*P*[*D*]) and the Δ*K* values (Evanno *et al.* 2005) were visualized in the STRUCTURE HARVESTER Web application (Earl and von Holdt 2011). Following Bayesian clustering, the hierarchical distribution of genetic variation was characterized using an analysis of molecular variance (AMOVA). A three-level AMOVA was conducted in ARLEQUIN 3.11 and significance was obtained via 10 000 random permutations.

### Tests for genetic bottleneck model

The program Bottleneck v.1.2.02 (Cornuet and Luikart 1996) was used to evaluate whether the examined populations suffered a severe genetic bottleneck or had experienced recent reductions in their size. A Wilcoxon-signed rank test of heterozygosity excess was used to evaluate the significance of a potential bottleneck. The analysis was performed under three different models of microsatellite evolution: the SMM model, the infinite allele model (IAM) and the two-phases model of mutation (TPM) with parameters of 30 % multiple-step mutations and 70 % single-step mutations. In addition, the distribution of allele frequencies over all loci was examined for a ‘mode-shift’, which might indicate a bottlenecked population rather than a stable population.

## Results

### Allelic diversity and within-population genetic variation

The 13 nuclear microsatellite loci investigated were polymorphic, providing a total of 160 size variants. There was no significant linkage disequilibrium between pairs of loci across all populations (*P* > 0.01). The number of alleles per locus ranged from 3 (psyl25) to 40 (Spag7.14), with an average of 12.3 [**see Supporting Information—Table S2**]. The estimated frequency of null alleles for most of the loci was moderate (< 6 %, but generally < 2 %) with the exceptions occurring at three loci: psyl18 (8.7 %), Spag7.14 (8.3 %) and PtTX3107 (16.2 %) [**see Supporting Information—Table S2**].

The basic statistics for genetic variation within the populations are summarized in Table 1. More than five alleles were observed in each population with an average Ap = 6.08. The allelic richness measures obtained based on a minimum of 22 samples (*A*_R22_) were comparable in all investigated populations and ranged from 5.1 in the T1 population to 6.2 in the PL1 population. The mean effective number of alleles was Ae = 3.4. The lowest number of effective alleles was observed in the Tokat–Yıldızeli population from Turkey (T3, Ae = 2.9), whereas the highest number of effective alleles was found in the population from southwestern Poland (PL1, Ae = 3.8). Twenty private alleles were also detected among some of the studied populations, and their frequency ranged from Pa = 0.0 % to Pa = 23.1%. Similar levels of gene diversity were found in all populations, which ranged from 0.48 to 0.53. However, pine populations from the peripheral stands appear to show greater diversity (with an average of 0.52) in comparison with populations from central and northern Europe (with an average of 0.48). The level of the overall observed heterozygosity (Ho) per population (average = 0.45, range: 0.38–0.49) was similar for all populations and slightly lower than the level of expected heterozygosity (He) (average = 0.50, range: 0.44–0.54). The inbreeding coefficients ranged from 0.017 in the F2 population from Finland to 0.282 in the T5 population from Turkey, with an overall mean of 0.120. However, the inbreeding coefficients may be highly overvalued due to the presence of null alleles. Because of this and based on our previous results that showed that null alleles were present at all investigated loci [**see Supporting Information—Table S2**], we used the IIM approach (see M&M) to partition out their influence on *F*_isNull_ (inbreeding coefficients with null alleles correction) value. Recalculated values of inbreeding coefficients, taking into account the frequency of null alleles (*F*_isNull_), were much lower than those obtained previously and ranged from 0.014 to 0.081 with an average of 0.045. The values of *F*_isNull_ and *F*_is_ were significantly different from zero in all populations. However, it seems that deficiency of heterozygotes is mostly due to the presence of null alleles. Moreover, it should be noted that the highest values of both *F*_is_ and *F*_isNull_ were observed in peripheral pine populations from Turkey and Spain.

### Genetic differentiation between populations

The analysis of molecular variance (AMOVA) based on the number of different alleles (*F*_ST_) and the sum of the squared size differences (*R*_ST_) showed that differentiation between Scots pine populations was low but significant (*F*_ST_ = 0.035, *R*_ST_ = 0.032; *P*< 0.0001). The majority of the variance was found within populations (Table 2). The global *F*_ST_ values, estimated both with and without the ENA correction, was *F*_ST_ = 0.035 and F_ST_ENA = 0.037, respectively. The similarity of these values implies that the presence of null alleles is not a significant factor affecting the level of genetic differentiation. The results of genetic differentiation among populations within the studied geographical regions are presented in Table 2. The greatest differentiation was found between populations from Turkey and Spain, whereas the within-region *F*_ST_ values obtained for the Balkans, Poland and Finland were not statistically significant. Most of the pairwise *F*_ST_ population values were significant (*P* < 0.001) [**see Supporting Information—Table S3**]. The greatest difference (0.11) was between the T4 population from Çatacık in Turkey and the H2 population from the Sierra de Neila in Spain, and the lowest (*F*_ST_ < 0.01) was between the PL2 population located in southern Poland and the PL4 population located in northern Poland.

**Table 2.** Analysis of molecular variance (AMOVA) at 13 *n*SSR loci. (a) Assuming no population structure; (b) among populations within geographical regions; (c) assuming population structure as defined by allelic frequency spectra and Bayesian assignment tests (Table 3).

Source of variation		d.f.	Sum of squares	Variance components	Percentage of variation	*P*
(a)						
Among populations	F_ST_	23	240.861	0.12172	3.55	< 0.0001
	R_ST_		48 995.78	25.16	3.50	< 0.0001
Within populations	F_ST_	652	4318.50	3.31	96.45	< 0.0001
	R_ST_		903 431.90	693.43	96.50	< 0.0001
(b)						
Turkey	F_ST_	5	46.48	0.11	3.25	< 0.0001
		100	954.49	3.37	96.75	< 0.0001
Spain	F_ST_	4	34.31	0.08	2.38	< 0.0001
		124	977.59	3.29	97.62	< 0.0001
Balkans	F_ST_	2	8.47	0.01	0.22	0.9984
		51	556.43	3.49	99.78	< 0.0001
Poland	F_ST_	3	18.59	0.03	1.13	0.2424
		95	812.06	3.24	98.87	< 0.0001
Finland	F_ST_	3	12.60	0.01	0.49	0.9918
		74	622.43	3.21	99.51	< 0.0001
(c)						
Among groups	F_ST_	5	120.93	0.08	2.51	< 0.0001
Among populations within groups		18	109.37	0.04	1.36	< 0.0001
Among individuals within populations		652	2327.52	0.35	10.64	< 0.0001
Within individuals		676	1932.00	2.85	85.48	< 0.0001

The permutation test, by which global *R*_ST_ was compared against the distribution of 10 000 p*R*_ST_ values, did not detect a significant difference between these parameters (*R*_ST _= 0.032; p*R*_ST _= 0.025; CIpR_ST_ 95 % = 0.01–0.04; p_H1_: *R*_ST _>_ _p*R*_ST_ = 0.164). This suggests an absence of phylogeographic structure and that gene flow is high compared with the mutation rate. However, the results of the Mantel test correlation between genetic distance and the logarithm of geographic distance among all *P. sylvestris* populations indicated that the genetic diversity is structured in geographic space (*R* = 0.39, *P* < 0.05). The strongest correlation was found along a longitudinal transect among populations from Anatolia and the Iberian Peninsula (*R *= 0.73, *P* < 0.05). The spatial genetic structure among all populations was rather weak, whereas only 15 % (*R*_2_ = 0.152) of the genetic divergence was explained by geographical distance. The strong structure (53 % of the genetic divergence could be explained by geographical distance) found among south peripheral populations is most likely due to the presence of geographical barriers along Mediterranean basin transects which intensify the effects of the process of isolation by distance (IBD).

### Population clustering and phylogenetic relationships

**Figure 2.**
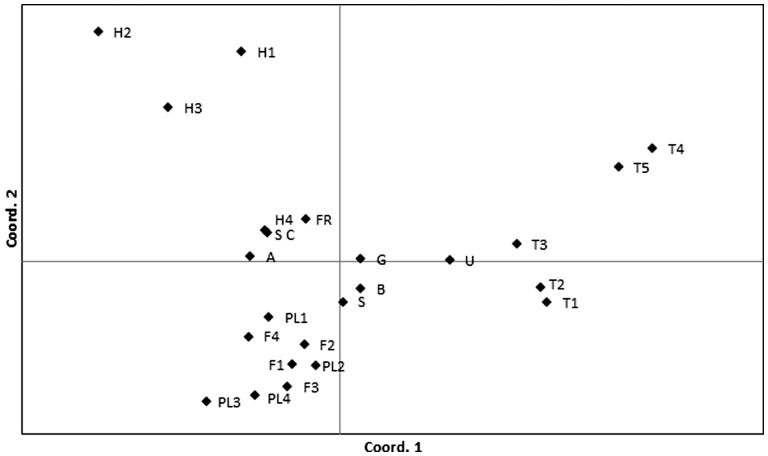
Principal Coordinate Analysis (PCoA) based on pairwise population F_ST_ENA values. Acronyms of Scots pine populations are listed in **Supporting Information—Table S1**.

The genetic structure of the Scots pine populations based on the pairwise F_ST_ENA matrix is illustrated in the PCoA plot (Fig. 2). The east–west subdivision of the southernmost peripheral populations was clearly shown by the first coordinate, which explained more than 25 % of the variation. The second variable (second coordinate), which was responsible for more than 20 % of the total variation, separated the pine populations from central and northern Europe from those in southern Eurasia. Moreover, according to the *F*_ST_ values obtained for each geographical region (Table 2), populations from both Turkey and Spain formed a much more heterogeneous group in comparison with populations from the Balkans and central and northern Europe.

The relationships between populations are illustrated in **Supporting Information—Figure S1**, which shows the phylogenetic tree based on the NJ method [**see Supporting Information—Figure S1**].

**Figure 3.**
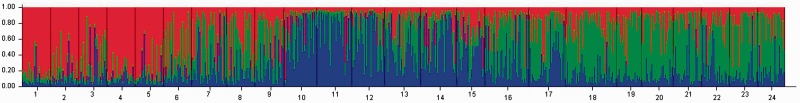
The STRUCTURE assignment of each individual to inferred genetic clusters (marked in different colours). The study populations are separated by thick black lines. Population numbers (1–24) are listed in Table 1 and **Supporting Information—Table S1**.

The Bayesian assignment of samples obtained with STRUCTURE indicated that three clusters (*K* = 3) provide the most probably representation of the overall genetic structure of the analysed Scots pine populations. The admixture proportions of each of the three gene pools (clusters) were estimated and differed among populations (Fig. 3). The highest frequency of cluster 1 (indicated in red) was found in populations from Turkey (T1, T2, T3, T4 and T5), with an average of 77.6 %, whereas cluster 2 (indicated in green) was most frequent in populations from Poland and Finland (PL1, PL2, PL3, PL4, F1, F2, F3 and F4), with a mean of 61.5%. Three populations from Spain (H1, H2 and H3) showed the highest frequencies of cluster 3 (indicated in blue; mean value of 69.1%). Pines from Ukraine, Balkans, north of Spain, Massif Central in France and Scotland were classified as mixed populations. However, the gene pool of the population from Ukraine exhibited a predominance of cluster 1, whereas populations from the Balkans showed a predominance of cluster 2. Gene pools from north of the Iberian Peninsula (A, H4) and the population from France exhibited a higher frequency of cluster 3. Clusters 2 and 3 contained the dominant frequencies in the gene pool of Scottish pine populations, with a higher frequency of cluster 2.

Based on these findings and the pairwise *F*_ST_ matrix [**see Supporting Information—Table S3**], an AMOVA analysis was conducted between six groups of populations. The largest group consisted of the populations from Turkey and Ukraine (Group 1). The next largest groups included populations from the Balkans (Group 2), southern Spain (Group 3), north of the Iberian Peninsula (populations H4 and A) and the population from the Massif Central in France (Group 4). Two other groups were formed by the population from Scotland (Group 5) and the populations from central and northern Europe (Group 6). The AMOVA results and the pairwise *F*_ST_ values between these groups of populations are presented in Tables 2 and 3, respectively. Significant genetic differentiation over short geographical distances was observed between the populations within Spain and Turkey (Table 2) but not between populations from the Balkans, Poland or Finland. The highest degree of genetic differentiation was found between southern Spain (Group 3) and Turkey and Ukraine (Group 1) and between those geographical areas and the rest of the populations (Table 3; **see Supporting Information—Table S3**).

**Table 3.** Genetic differentiation (F_ST_) between groups of Scots pine populations according to population grouping analysis and geographical location of populations (*P < 0.001).

	1	2	3	4	5
1. Turkey and Ukraine	0.000				
2. Greece, Serbia and Bulgaria	0.021*	0.000			
3. Southern Spain (H1, H2 and H3)	0.063*	0.039*	0.000		
4. Northern Spain (H4), Andorra and France	0.035*	0.017*	0.025*	0.000	
5. Scotland	0.041*	0.021*	0.029*	0.010*	0.000
6. Poland and Finland	0.035*	0.009*	0.042*	0.014*	0.016*

### Tests for genetic bottleneck model

Despite the fact that some of the investigated populations were characterized by lower genetic diversity, no evidence for a recent genetic bottleneck or reductions in effective population size under the two-phased mutation model (TPM), infinite allele mutation model (IAM) or stepwise mutation model (SMM) was detected. Moreover, the mode-shift test, based on the frequency distribution of alleles, demonstrated a typical L-shaped mode for all populations, which is consistent with a non-bottleneck model.

## Discussion

### Genetic variation and differentiation

In our study, we evaluated the neutral genetic variation and genetic structure of Scots pine populations from the core and peripheral distribution in Europe and Asia Minor based on variation in *n*SSR markers. All the 13 microsatellite loci studied were polymorphic with a mean of 12.3 alleles per locus. Considerably lower numbers of alleles were found at the psyl loci (an average of 6.1) compared with the Spag and PtTX loci (an average of 30.5 and 14.0, respectively). The most variable loci were Spag7.14, Spac11.4 and PtTX2146. Therefore, these loci appear to be the most informative for population genetics studies of *P. sylvestris* that require a high resolution of markers for fine-scale analysis. Consistent with earlier studies on the genetic variation of conifer species, we found that the analysed loci contained some level of null alleles (Liewlaksaneeyanawin *et al.* 2004; Belletti *et al.* 2012; Sebastiani *et al.* 2012). However, as evidenced by our dataset, they did not significantly affect the observed patterns of variation across the studied populations.

Our data show that most of the genetic variation is located within the Scots pine populations. The level of genetic variation, as measured by basic statistics, was high and very similar across populations (Table 1) which is typical to woody species (Hamrick *et al.* 1992). The levels of observed heterozygosity were only slightly less than the values of unbiased expected heterozygosity and were primarily due to the presence of null alleles. These findings indicate that there is no significant allele frequency disequilibrium in any of the studied Scots pine populations. There were, however, some noticeable differences in the parameters describing the within-population genetic variation of peripheral populations vs. populations from the core distribution. In general, the peripheral populations had higher frequencies of private alleles, with the exception of the Balkan populations. An excess of private alleles was also observed in the peripheral Scots pine populations from Spain and Italy based on an analysis of allozyme and *n*SSR loci (Belletti *et al.* 2012; Prus-Głowacki *et al.* 2012). Those populations had also higher inbreeding coefficients in comparison with populations from the Balkans, Poland and Finland. The results of this study indicate isolation and probably a limited effective population size in the peripheral populations from Turkey (T2, T3, T4 and T5) and the south of Spain (H1, H2 and H3). The stronger isolation of these pine populations from the south regions apparently restricts gene flow and seems to cause a more distinct pattern of geographical variation in these studied regions. This conclusion is supported by the results of the Mantel test correlation analysis, which is based on genetic and geographical distance. We found that there was strong spatial structure and overall among-population differentiation between southern peripheral populations. This high differentiation most likely results from action of the processes of isolation-by-distance and genetic drift, which are most effective in isolated stands. It must be pointed, however, that the isolation did not have a strong negative impact on the level of within population genetic variation found in the peripheral pine populations. Similarly relationship was already demonstrated in the case of other plant species living in isolation, as for example in the Cheddar pink (Putz *et al.* 2015). Our results also show contrasting patterns of genetic variation between populations located within distinct geographical areas. Populations within the Iberian and Anatolian Peninsulas are separated by relatively short geographical distances but showed much higher divergence compared with populations from northern Europe and the Balkans, which are separated by several thousand kilometres. In some previous studies, relatively high genetic diversity (*F*_ST_ = 0.058) was found within the Scots pine natural range in Italy (Belletti *et al.* 2012) as compared to differentiation between populations from the Scandinavian region (*F*_ST_ = 0.02, Karhu *et al.*1996). Similarly, a high level of differentiation between *P.* *sylvestris* populations from mountain regions in Spain in relation to other European populations has been found (Prus-Głowacki and Stephan 1993; Prus-Głowacki *et al.* 2003). Moreover, a high level of differentiation between populations of Norway spruce from Bulgarian mountains in comparison to populations from other regions of natural distribution of this species in Europe was noted by Tollefsrud *et al.* (2008). Mountain regions of southern Europe are assumed to have provided particularly suitable habitats for species survival during last glacial period, because they allowed species to respond to climate oscillations using the altitudinal gradient (Hewitt 1996). Our results support the hypothesis of a possible long-lasting isolation of pinewoods in separate Iberian refugia (Rubiales *et al.* 2010) and some pine populations in separate refugial areas within the Anatolian Peninsula. Moreover these findings suggest that the homogenizing effect of gene flow via pollen dispersal on gene pool variation across geographical ranges may be limited.

### The genetic relationships between populations

Although only a small percentage of the total variation was due to differentiation among populations (approximately 4%), we found clear signals of population substructuring at *n*SSR loci within the Scots pine distribution. Based on the analysis of allelic frequency spectra and population assignment methods, we could distinguish several groups of populations from distinct geographical locations. Five groups of populations are clearly represented at the PCoA plot where Scottish population is grouped with the populations from north of Iberia Peninsula and Massif Central in France. However taking into account the admixture proportions of each of the three gene pools in the analysed populations and the result of phylogenic relationship analysis, we decided to separate the population from Scotland as a distinct group because it could not be clearly attributed to any of the designated previously groups of populations. As a result, the AMOVA analysis was conducted between six groups of populations. The east–west subdivision was clearly shown between southernmost groups of populations from Turkey (Group 1) and Spain (Group 3). The differentiation between the Iberian and Anatolian populations was also the greatest based on the morphological and anatomical traits of needles from Scots pines in the Mediterranean Basin (Jasińska *et al.* 2014). Phenotypic and genetic differentiation among populations from these regions support the isolation of *P. sylvestris* in the southern European and Anatolian mountain regions and suggest that these populations are characterized by a different population history. In our study, the populations from Turkey and the south of Spain were significantly different from populations in the Balkans (Group 2), north of the Iberian Peninsula and the Massif Central (Group 4), Scotland (Group 5) and Poland and Finland (Group 6). In some previous mitochondrial DNA studies, unique haplotypes not observed in other parts of Europe were detected in Spanish and Turkish populations (Cheddadi *et al.* 2006; Naydenov *et al.* 2007; Pyhäjärvi *et al.* 2008). Our data show a divergence between populations within the Iberian Peninsula and indicate that southern populations have distinct gene pools compared with northern Spain and Andora populations. The latter population has probably contributed to the recolonization of Europe (or was admixed) after the last glacial maximum, as evidenced by genetic variation similar to populations from Massif Central and the Balkans and minor differentiation from populations within the continuous distribution. The subdivision of populations from the Iberian Peninsula has also been suggested by the analysis of biochemical and molecular markers (Prus-Głowacki *et al.* 2003, 2012; Robledo-Arnuncio *et al.* 2005).

The position of the Scottish populations is not straightforward. Scottish populations showed some similarity to groups from northern Spain and the Massif Central and populations from central and northern Europe. Previous pollen, allozyme and monoterpene studies suggested a west/east population subdivision within Scotland, reflecting different origins of the populations contributing to the postglacial colonization (Birks 1989; Kinloch *et al.* 1986; Sinclair *et al.* 1998). Based on this finding, two hypotheses have been put forth to explain this phenomenon. Kinloch *et al.* (1986) suggested that the western Scotland pine populations are relicts and might have survived the last glacial maximum in some region of the British Isles because they show relatively little genetic affinity to pines from the continuous range. The eastern populations were likely admixed with populations of continental origin or derived from that distribution. Scottish populations also show high levels of genetic polymorphism at nuclear gene loci and patterns of allelic frequency incompatible with a simple model of population expansion from mainland locations (Wachowiak *et al.* 2011). Given predictions regarding the extent of the British ice sheet during the last glaciation (Clark *et al.* 2012) and the geographic distribution of some unique mitochondrial haplotypes, it appears more probable that colonization of the Scottish Highlands occurred from at least two different sources (Sinclair *et al.* 1998; Soranzo *et al.* 2000). Our data show that the composition of the gene pool of the Scottish population has characteristics in common with populations from both central Europe and north of the Iberian Peninsula. We also found high frequencies of private alleles in this population. However, our data are too limited to provide any definitive answer regarding the source of the probable Scots pine admixture in the Scottish Highlands.

The Scots pine populations from the continuous range in Poland and Finland were characterized by the most homogeneous gene pools, and differentiation between populations from those regions was not statistically significant. The low level of genetic differentiation between Scots pine populations from central and northern Europe was also observed for isozymes (Prus-Głowacki *et al.* 2012; Sannikov and Petrova 2012) and nucleotide diversity at nuclear gene loci (Wachowiak *et al.* 2014). These results imply free gene exchange among those populations and that they probably share a common postglacial history. Moreover, our data show that populations from the Balkans share gene pools with populations from central and northern Europe. More challenging is the identification of putative source populations for the recolonization of pines following the last glacial maximum. None of the analysed populations in our study showed exceptionally high genetic variation or evidence for recent bottlenecks following the patterns of nucleotide sequence variation at nuclear gene loci (Pyhäjärvi *et al.* 2007; Wachowiak *et al.* 2009; Kujala and Savolainen 2012). Taking into account findings about genetic consequences of glacial isolation and postglacial colonization on the genetic structure of other cold tolerant tree species e.g. silver birch (*Betula pendula*) and Norway spruce (*Picea abies*) which at present co-occur with Scots pine across large parts of its natural habitat, we can conclude that Europe most likely was re-occupied by at least three main waves of recolonization. The source populations could have their origins in the regions of Alps, Balkans and east refugia with origin at intermediate latitude in Moscow region (Palmé *et al.* 2003; Willis and van Andel 2004; Maliouchenko *et al.* 2007; Tollefsrud *et al.* 2008; Parducci *et al.* 2012). These several distinct lineages of colonization might have mixed in central Europe as suggested based on colonization routes of Norway spruce (Dering and Lewandowski 2009). However, according to results obtained by Parducci *et al.* (2012) which suggest that conifer trees might have also survived the last glaciation in the ice-free refugia of Scandinavia, it appears that detailed studies of populations from Scandinavia and the eastern distribution in Asia, including regions of Moscow (Buchovska *et al.* 2013), are needed to test the possible recolonization trajectories of the species to the central Europe.

## Conclusions

Our data show that isolated populations from southern Eurasia show a high genetic divergence over a short geographical distance, which contrasts with the pattern of variation in populations from the northern part of Europe. A clear subdivision was found between populations from distinct parts of the Eurasian distribution. With the exception of the southernmost stands, populations from the central and northern parts of the studied distribution range showed some genetic similarity that may reflect their shared postglacial history or effective admixture between populations of different origin. High-resolution *mt*DNA markers dispersed by seeds across small geographic areas in pines would be needed to verify migration trajectories and the location of source populations for the species’ postglacial recolonization.

## Sources of Funding

This work was financially supported by the Polish National Science Centre (DEC-2012/05/E/NZ9/03476).

## Contributions by the Authors

W.W. and B.W. designed and conceptualized the study. B.W. and M.L. collected and analysed the data. B.W. wrote the manuscript; W.W. assisted in drafting the manuscript; B.W. and W.W. critically reviewed and revised the manuscript for content; all authors read and approved the final manuscript.

## Conflict of Interest Statement

None declared.

## Supplementary Material

Supplementary Data

## Supporting Information

The following additional information is available in the online version of this article —

 **Table S1.** Populations of *Pinus sylvestris* used in this study.

 **Table S2.** Descriptive statistics for the thirteen nuclear microsatellite loci used in this study. Al, number of alleles; A_NullFreq_, mean frequency of null alleles.

 **Table S3.** Pairwise F_ST_ENA matrix for 24 Scots pine populations (*p < 0.001).

 **Figure S1.** Phylogenetic tree of 24 Scots pine populations based on Nei's standard genetic distance (D_ST_) at 13 *n*SSR loci (1000 bootstraps) using the neighbour-joining method (NJ).
