# Supplementary material for: Contrasting patterns of genetic variation in core and peripheral populations of highly outcrossing and wind pollinated forest tree species
Source: AoB Plants. 2016 Aug 6;8:plw054. doi: 10.1093/aobpla/plw054 (PMC5018396; doi:10.1093/aobpla/plw054)
Supplement: Supplementary Data [file supp_8_plw054_index.html]

Contrasting patterns of genetic variation in core and peripheral populations of highly outcrossing and wind pollinated forest tree species — Supplementary Data 

# Contrasting patterns of genetic variation in core and peripheral populations of highly outcrossing and wind pollinated forest tree species

## Supplementary Data

files

- Supplementary Data - zip file
