# Supplementary material for: Contrasting patterns of genetic variation in core and peripheral populations of highly outcrossing and wind pollinated forest tree species
Source: AoB Plants. 2016 Aug 6;8:plw054. doi: 10.1093/aobpla/plw054 (PMC5018396; doi:10.1093/aobpla/plw054)

**Table S1.** Populations of *Pinus sylvestris* used in this study.

| Code | Location of population | | Longitude | Latitude | | Altitude |
| --- | --- | --- | --- | --- | --- | --- |
| T1 | Turkey | Şavşat- Ardahan | E 42.43 | | N 41.23 | 1700 |
| T2 |  | Sakaltutan Geçidi. S from Şiran | E 39.05 | | N 39.87 | 2010 |
| T3 |  | Tokat-Yıldızeli | E 36.52 | | N 39.96 | 1579 |
| T4 |  | Çatacık | E 31.11 | | N 39.96 | 1619 |
| T5 |  | Bayabat-Sinop | E 34.83 | | N 41.64 | 1228 |
| U | Ukraine | Crimea. Yalta | E 34.20 | | N 44.55 | 1380 |
| G | Greece | Ano Vrandou | E 23.65 | | N 41.31 | 1350 |
| B | Bulgaria | Pirin. Bansko-Razlog | E 23.36 | | N 41.88 | 1076 |
| S | Serbia | Divčibare Mts | E 19.99 | | N 44.10 | 957 |
| H1 | Spain | Sierra de Gúdar. Valldelinares | W 00.61 | | N 40.38 | 1950 |
| H2 |  | Sierra de Neila | W 03.01 | | N 42.05 | 1400 |
| H3 |  | Puerto de Navafría | W 03.81 | | N 40.98 | 1800 |
| H4 |  | Pyrenees. below Tunel de Viella | E 01.57 | | N 42.52 | 1500 |
| A | Andorra | St. Miguel d’Engolasters | E 00.77 | | N 42.67 | 1550 |
| F | France | Forêt Domaniale | E 02.02 | | N 45.66 | 800 |
| SC | Scotland | Shieldaig  Glen Tanar  Rothiemurcys  Glen Affric  Black Wood of Rannoch | W 5.38 | | N 57.32 | 81 |
|  |  |  | W 2.51 | | N 57.21 | 160 |
|  |  |  | W 3.46 | | N 57.81 | 318 |
|  |  |  | W 4.55 | | N 57.16 | 256 |
|  |  |  | W 4.19 | | N 56.40 | 275 |
|  |  | Glen Loy | W 5.13 | | N 56.91 | 170 |
| PL1 | south- western Poland | Chojnik | E 15.64 | | N 50.83 | 600 |
|  |  | Szczeliniec | E 16.34 | | N 50.48 | 900 |
| PL2 | southern Poland | Rezerwat "Pusta Wielka" | E 20.82 | | N 49.40 | 1000 |
|  |  | Tatry. Koryciska Wielkie | E 19.83 | | N 49.27 | 1000 |
|  |  | Pieniński Park Narodowy | E 20.36 | | N 49.42 | 800 |
|  |  | Torfowiska Tarnawa | E 22.49 | | N 49.10 | 650 |
| PL3 | eastern Poland | Hajnówka | E 23.58 | | N 52.74 | 165 |
|  |  | Rezerwat Liski | E 22.82 | | N 51.95 | 150 |
| PL4 | northern Poland | WDN Woziwoda | E 17.91 | | N 53.67 | 120 |
|  |  | Rezerwat Tabórz | E 20.04 | | N 53.77 | 115 |
|  |  | Miłomłyn | E 19.84 | | N 53.76 | 100 |
| F1 | Finland | Joutsa | E 26.14 | | N 61.74 | 125 |
| F2 |  | Area near Temmes and Tyrnävä | E 25.71 | | N 64.69 | 64 |
| F3 |  | Area near Rovaniemi | E 26.21 | | N 66.57 | 120 |
| F4 |  | Area near Kielajoki | E 29.07 | | N 69.65 | 100 |

**Table S2.** Descriptive statistics for the thirteen nuclear microsatellite loci used in this study. Al, number of alleles; A_NullFreq_, mean frequency of null alleles.

| **Locus** | **Primers sequence 5'–3'** | **Repeated motif** | **Size range** | **Al** | **A_NullFreq_** | **References** |
| --- | --- | --- | --- | --- | --- | --- |
|  |  |  |  |  |  |  |
| psyl2 | F: TTGCTTTTGCAGAACATTCG | (gct)5 | 200-221 | 6 | 2.7% | Sebastiani et al. (2012) |
|  | R: GTCCTGCAGGCAATCAAAAT |  |  |  |  |  |
| psyl18 | F: ACTACCTGGCATTCGTCCTG | (gca)7 | 288-306 | 7 | 8.7% | Sebastiani et al. (2012) |
|  | R: GGATCTGGTCCATTTCGTGT |  |  |  |  |  |
| psyl25 | F: CAGCACGCGTTCTTTGTATC | (gca)5 | 212-218 | 3 | 1.0% | Sebastiani et al. (2012) |
|  | R: ACCGTTGCTCGTTGTCTTCT |  |  |  |  |  |
| psyl36 | F: TATCATCGAGAGCCCCAAAA | (gtc)7 | 247-265 | 8 | 5.3% | Sebastiani et al. (2012) |
|  | R: GAAAGGCGAAAGCAAAAGTG |  |  |  |  |  |
| psyl42 | F: CAACTTCAGCCTTGCAACAA | (tc)9 | 168-180 | 7 | 1.9% | Sebastiani et al. (2012) |
|  | R: CGACTTCATTTGGAACACCA |  |  |  |  |  |
| psyl44 | F: TCCAAGTTCGGTTCCTTGTC | (cgg)5 | 169-178 | 4 | 1.5% | Sebastiani et al. (2012) |
|  | R: GACACGATGGATTCCCTGAT |  |  |  |  |  |
| psyl57 | F: CCCCACATCTCTACAGTCCAA | (acc)7 | 186-207 | 8 | 0.9% | Sebastiani et al. (2012) |
|  | R: TGCTCTTGGATTTGTTGCTG |  |  |  |  |  |
| Spag7.14 | F: TTCGTAGGACTAAAAATGTGTG | (tg)17(ag)21 | 176-258 | 40 | 8.3% | Soranzo et al. (1998) |
|  | R: CAAAGTGGATTTTGACCG |  |  |  |  |  |
| Spac11.4 | F: TCACAAAACACGTGATTCACA | (at)5(gt)19 | 130-170 | 21 | 1.8% | Soranzo et al. (1998) |
|  | R: GAAAATAGCCCTGTGTGAGACA |  |  |  |  |  |
| PtTX2146 | F:CCTGGGGATTTGGATTGG | (gct)4gcc(gtc)7gcc(gct)8 | 174-252 | 23 | 2.8% | Elsik et al. (2000) |
|  | R: ATATTTTCCTTGCCCCTTCCAGAC |  |  |  |  |  |
| PtTX3025 | F: CACGCTGTATAATAACAATCTA | (caa)10 | 260-299 | 11 | 3.6% | Elsik et al. (2000) |
|  | R: TTCTATATTCGCTTTTAGTTTC |  |  |  |  |  |
| PtTX3107 | F: AAACAAGCCCACATCGTCAATC | (CAT)14 | 153-183 | 10 | 16.2% | Elsik and Williams (2001) |
|  | R: TCCCCTGGATCTGAGGA |  |  |  |  |  |
| PtTX4011 | F: GGTAACATTGGGAAAACACTCA | (CA)20 | 231-281 | 12 | 5.0% | Zhou et al. (2002) |
|  | R: TTAACCATCTATGCCAATCACTT |  |  |  |  |  |

|  | T1 | T2 | T3 | T4 | T5 | U | G | B | S | H1 | H2 | H3 | A | H4 | FR | S C | PL1 | PL2 | PL3 | PL4 | F1 | F2 | F3 |
| --- | --- | --- | --- | --- | --- | --- | --- | --- | --- | --- | --- | --- | --- | --- | --- | --- | --- | --- | --- | --- | --- | --- | --- |
| T2 | 0.01 |  |  |  |  |  |  |  |  |  |  |  |  |  |  |  |  |  |  |  |  |  |  |
| T3 | 0.03* | 0.02* |  |  |  |  |  |  |  |  |  |  |  |  |  |  |  |  |  |  |  |  |  |
| T4 | 0.04* | 0.03* | 0.05* |  |  |  |  |  |  |  |  |  |  |  |  |  |  |  |  |  |  |  |  |
| T5 | 0.03* | 0.04* | 0.04* | 0.01 |  |  |  |  |  |  |  |  |  |  |  |  |  |  |  |  |  |  |  |
| U | 0.04* | 0.05* | 0.04* | 0.06* | 0.04* |  |  |  |  |  |  |  |  |  |  |  |  |  |  |  |  |  |  |
| G | 0.03* | 0.03* | 0.03* | 0.04* | 0.04* | 0.03* |  |  |  |  |  |  |  |  |  |  |  |  |  |  |  |  |  |
| B | 0.03* | 0.03* | 0.03* | 0.06* | 0.05* | 0.03* | 0.00 |  |  |  |  |  |  |  |  |  |  |  |  |  |  |  |  |
| S | 0.03* | 0.03* | 0.03* | 0.06* | 0.05* | 0.02* | 0.01 | 0.01 |  |  |  |  |  |  |  |  |  |  |  |  |  |  |  |
| H1 | 0.06* | 0.06* | 0.06* | 0.07* | 0.06* | 0.05* | 0.03* | 0.04* | 0.04* |  |  |  |  |  |  |  |  |  |  |  |  |  |  |
| H2 | 0.09* | 0.10* | 0.09* | 0.11* | 0.10* | 0.07* | 0.05* | 0.06* | 0.06* | 0.01 |  |  |  |  |  |  |  |  |  |  |  |  |  |
| H3 | 0.07* | 0.08* | 0.06* | 0.10* | 0.09* | 0.07* | 0.04* | 0.04* | 0.05* | 0.02* | 0.02 |  |  |  |  |  |  |  |  |  |  |  |  |
| A | 0.05* | 0.05* | 0.05* | 0.08* | 0.07* | 0.04* | 0.02* | 0.03* | 0.02* | 0.03* | 0.04* | 0.04* |  |  |  |  |  |  |  |  |  |  |  |
| H4 | 0.05* | 0.05* | 0.04* | 0.07* | 0.08* | 0.04* | 0.01* | 0.03* | 0.03* | 0.03* | 0.03* | 0.03* | 0.01 |  |  |  |  |  |  |  |  |  |  |
| FR | 0.04* | 0.04* | 0.05* | 0.05* | 0.05* | 0.03* | 0.01 | 0.03* | 0.01 | 0.02* | 0.03* | 0.04* | 0.01 | 0.01 |  |  |  |  |  |  |  |  |  |
| S C | 0.05* | 0.05* | 0.05* | 0.07* | 0.07* | 0.04* | 0.02* | 0.02* | 0.03* | 0.03* | 0.04* | 0.03* | 0.02* | 0.01 | 0.00 |  |  |  |  |  |  |  |  |
| PL1 | 0.04* | 0.06* | 0.06* | 0.07* | 0.07* | 0.03* | 0.01* | 0.02* | 0.02* | 0.04* | 0.06* | 0.05* | 0.02* | 0.02* | 0.02* | 0.02* |  |  |  |  |  |  |  |
| PL2 | 0.04* | 0.04* | 0.04* | 0.08* | 0.06* | 0.03* | 0.01* | 0.01 | 0.01 | 0.05* | 0.07* | 0.06* | 0.01 | 0.03* | 0.02* | 0.02* | 0.02 |  |  |  |  |  |  |
| PL3 | 0.05* | 0.06* | 0.07* | 0.11* | 0.09* | 0.04* | 0.03* | 0.02* | 0.02* | 0.06* | 0.06* | 0.07* | 0.02* | 0.05* | 0.03* | 0.04* | 0.03* | 0.01 |  |  |  |  |  |
| PL4 | 0.04* | 0.05* | 0.06* | 0.10* | 0.08* | 0.05* | 0.02* | 0.01 | 0.02* | 0.06* | 0.07* | 0.05* | 0.02* | 0.03* | 0.03* | 0.02* | 0.02 | 0.00 | 0.01 |  |  |  |  |
| F1 | 0.03* | 0.05* | 0.04* | 0.08* | 0.06* | 0.04* | 0.02* | 0.01 | 0.01 | 0.05* | 0.07* | 0.06* | 0.02* | 0.03* | 0.02* | 0.02 | 0.02* | 0.01 | 0.02* | 0.01 |  |  |  |
| F2 | 0.03* | 0.04* | 0.05* | 0.07* | 0.06* | 0.02* | 0.01 | 0.01 | 0.01 | 0.04* | 0.06* | 0.04* | 0.02* | 0.03* | 0.02* | 0.02* | 0.02* | 0.01 | 0.01 | 0.00 | 0.01 |  |  |
| F3 | 0.04* | 0.05* | 0.05* | 0.08* | 0.07* | 0.03* | 0.01 | 0.01 | 0.01 | 0.06* | 0.07* | 0.05* | 0.02* | 0.03* | 0.02* | 0.02 | 0.01 | 0.01 | 0.02* | 0.01 | 0.00 | 0.01 |  |
| F4 | 0.04* | 0.05* | 0.06* | 0.08* | 0.07* | 0.03* | 0.01* | 0.01 | 0.02* | 0.04* | 0.05* | 0.04* | 0.01* | 0.03* | 0.01 | 0.02* | 0.01 | 0.01 | 0.02* | 0.01 | 0.01 | 0.00 | 0.01 |

**Table S3.** Pairwise F_ST_ENA matrix for 24 Scots pine populations (*p < 0.001).

**Figure S1.** Phylogenetic tree of 24 Scots pine populations based on Nei's standard genetic distance (D_ST_) at 13 *n*SSR loci (1000 bootstraps) using the neighbor-joining method (NJ).


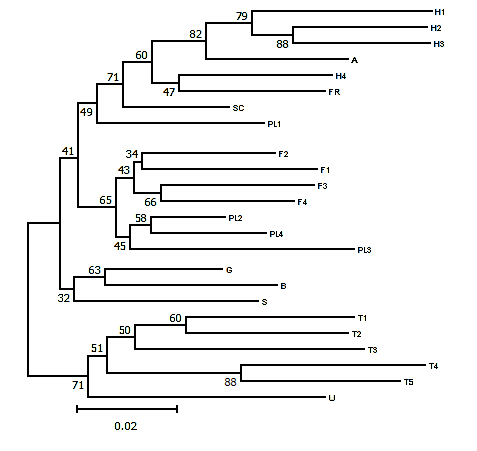

Supplement: Supplementary Data [file supp_plw054_Supplementary_files.zip › aobplants-16028-s01.docx]
