# Supplementary material for: Contrasting patterns of genetic variation in core and peripheral populations of highly outcrossing and wind pollinated forest tree species
Source: AoB Plants. 2016 Aug 6;8:plw054. doi: 10.1093/aobpla/plw054 (PMC5018396; doi:10.1093/aobpla/plw054)
Supplement: Supplementary Data [file supp_plw054_Supplementary_files.zip › aobplants-16028-s03.doc]

# OPEN ACCESS – RESEARCH ARTICLE;

# Contrasting patterns of genetic variation in core and peripheral populations of highly outcrossing and wind pollinated forest tree species

### Błażej Wójkiewicz^1,^*, Monika Litkowiec^1^ and Witold Wachowiak^1,2^

^1^Institute of Dendrology, Polish Academy of Sciences, Parkowa 5, 62-035 Kórnik, Poland.

^2^Institute of Environmental Biology, Faculty of Biology, Adam Mickiewicz University, Umultowska 89, 61-614 Poznań, Poland

*Corresponding author

Corresponding author’s e-mail address: bwojkiew@man.poznan.pl

Running head: Genetic variation in Scots pine
