# Supplementary material for: Contrasting patterns of genetic variation in core and peripheral populations of highly outcrossing and wind pollinated forest tree species
Source: AoB Plants. 2016 Aug 6;8:plw054. doi: 10.1093/aobpla/plw054 (PMC5018396; doi:10.1093/aobpla/plw054)
Supplement: Supplementary Data [file supp_plw054_Supplementary_files.zip › aobplants-16028-s02.docx]

Dear Editor,

We would like to submit the final version of the manuscript: Contrasting patterns of genetic variation in core and peripheral populations of highly outcrossing and wind pollinated forest tree species.

As requested, we changed or modified all the places that were listed in the review letter and marked by Managing Editor Gail Rice in the attached file. This also includes the addition of three sections (Sources of Funding; Contributions of Authors; and Conflicts of Interest) to our revised version of the manuscript.

Below we append a few items that can be used to promote paper on-line.

In the name of all co-authors, I would like you to accept the revised version of the manuscript for publication.

Sincerely Yours,

Błażej Wójkiewicz

1. Topics selected from TOPIC LIST that best categorize our paper:
   - Genetics & Genomics
2. Promotional statement

This study of nuclear microsatellite loci demonstrates genetic relationships between twenty four Scots pine populations from Europe and Asia Minor. The analysed populations were assigned to several groups that corresponded to the geographical regions of their occurrence. Significant differentiation over short geographical distances was observed between isolated populations within the Iberian and Anatolian Peninsulas (Asia Minor), which contrasted with the absence of genetic differentiation between distant populations, for instance in central and northern Europe. Despite the signs of isolation found in peripheral stands, a similar level of genetic variation and no evidence of a recent bottleneck was found across the populations.

The email list:

[borkrys@man.poznan.pl](mailto:borkrys@man.poznan.pl),

[borata@man.poznan.pl](mailto:borata@man.poznan.pl),

[alew@man.poznan.pl](mailto:alew@man.poznan.pl),

[J.Nowakowska@ibles.waw.pl](mailto:J.Nowakowska@ibles.waw.pl),

[ireko@amu.edu.pl](mailto:ireko@amu.edu.pl),

[prusw@amu.edu.pl](mailto:prusw@amu.edu.pl),

[lechurb@amu.edu.pl](mailto:lechurb@vp.pl)

3. There is no Special Issue for which our paper could be classified.

Image: (OR USE FIGURE 1 FROM MS)

**Figure S1.** Phylogenetic tree of 24 Scots pine populations based on Nei's standard genetic distance (D_ST_) at 13 *n*SSR loci (1000 bootstraps) using the neighbor-joining method (NJ).
